# Presence and Characterisation of Porcine Respirovirus 1 (PRV1) in Northern Italy

**DOI:** 10.3390/pathogens13010085

**Published:** 2024-01-18

**Authors:** Enrica Sozzi, Gabriele Leo, Cristina Bertasio, Giovanni Loris Alborali, Cristian Salogni, Matteo Tonni, Nicoletta Formenti, Davide Lelli, Ana Moreno, Tiziana Trogu, Sabrina Canziani, Clara Tolini, Monica Pierangela Cerioli, Antonio Lavazza

**Affiliations:** Istituto Zooprofilattico Sperimentale della Lombardia e dell’Emilia Romagna “Bruno Ubertini” (IZSLER), Via Antonio Bianchi 7/9, 25124 Brescia, Italy; gabriele.leo@izsler.it (G.L.); cristina.bertasio@izsler.it (C.B.); giovanni.alborali@izsler.it (G.L.A.); cristian.salogni@izsler.it (C.S.); matteo.tonni@izsler.it (M.T.); nicoletta.formenti@izsler.it (N.F.); davide.lelli@izsler.it (D.L.); anamaria.morenomartin@izsler.it (A.M.); tiziana.trogu@izsler.it (T.T.); sabrina.canziani@izsler.it (S.C.); clara.tolini@izsler.it (C.T.); monicapierangela.cerioli@izsler.it (M.P.C.); antonio.lavazza@izsler.it (A.L.)

**Keywords:** Porcine Respirovirus, phylogenetic analysis, epidemiology detection

## Abstract

Porcine Respirovirus 1 (PRV1) is an enveloped, single-stranded, negative-sense RNA virus belonging to the genus Respirovirus within the Paramyxoviridae family. Since its first detection in China in 2013, PRV1 has been identified in several American and European countries. Although its pathogenicity is uncertain, recent studies have suggested that it may play a role in the Porcine Respiratory Disease Complex (PRDC) because of its capacity to replicate in the upper and lower respiratory tracts. This study aimed to determine the spread of PRV1 in Northern Italy and the phylogeny of the isolates. Therefore, PRV1 was investigated using real-time RT-PCR in 902 samples collected from September 2022 to September 2023 from pigs with respiratory symptoms in North Italy. Fourteen (1.55%) samples tested as PRV1-positive. The full-length fusion (F) gene, which codifies for a major surface protein, was amplified and used for phylogenetic analysis to help carry out molecular epidemiological studies on this virus. In addition, swine influenza virus (SIV) and porcine reproductive and respiratory syndrome virus (PRRSV) infections were detected in most of the PRV1-positive samples. In conclusion, we report the detection of PRV1 in Italy and discuss its potential role as a co-factor in causing the Porcine Respiratory Disease Complex.

## 1. Introduction

Porcine Respirovirus 1 (PRV1) belongs to the Paramyxoviridae family and is an enveloped, single-stranded, negative-sense RNA virus. The genome, approximately 15 kb in length, encodes for six major proteins (3′-N-P-M-F-HN-L-5′) and three accessory proteins (V, W and C) [1]. Haemagglutinin–neuraminidase (HN) and fusion (F) glycoproteins on the viral surface play a role in the host cell infection process. The F and HN proteins are involved in receptor binding, present neutralising epitopes, and are genetically diverse.

In the last forty years, various paramyxoviruses have been identified in pigs, such as La Piedad Michoacan paramyxovirus (LPMV) [2], porcine rubulavirus (PoRV) [3], Menangle virus (MenV) [4], parainfluenza virus 3 (PIV3) [5], and porcine PIV5 (pPIV5) [6]. In 2013, PRV1 was first detected in dead pigs in Hong Kong, China [1]. Phylogenetic analysis revealed that PRV1 is closely related to human respirovirus 1 (HRV1) and murine respirovirus 1 (Sendai virus-SenV) and was then classified within the respirovirus genus [1,7]. Since its discovery, PRV1 has been detected in America and Europe, indicating its global spread [7,8,9,10,11].

PRV1 has been isolated in pigs with respiratory symptoms (cough and nasal discharge). Although its primary pathogenicity remains undefined, recent studies confirmed its potential role in causing the Porcine Respiratory Disease Complex (PRDC) in synergy with other viruses due to the ability to replicate in the upper and lower airways [11,12].

Northern Italy is an intensive pig-rearing area that could provide useful information on the circulation of PRV1. This study aimed to verify the circulation of PRV1 in this area and to genetically characterise PRV1-positive samples by determining the viral phylogeny. Phylogenetic analysis was conducted on the F gene, which is used for molecular epidemiological studies of this virus and is well established.

## 2. Materials and Methods

### 2.1. Sampling

In total, 902 samples (lungs, oral fluids, nasal swabs, and bronchial lavages) from pigs with respiratory symptoms were examined. They were all conferred within one year, from September 2022 to September 2023, to the IZSLER Diagnostic Laboratory in Brescia from farms located in three regions, namely Piedmont, Lombardy, and the Emilia Romagna, of Northern Italy. Most of the examined pigs were between four and six weeks of age, specifically from farms where clinical symptoms of respiratory diseases, including rhinitis with sneezing, coughing, and nasal discharge, were observed. In Table 1, we provide the details of the sample collection. After necropsy, a specific virological investigation and routine bacteriological examinations were planned and performed (Table 1).

Porcine diagnostic specimens were homogenised in minimum essential medium (MEM; Gibco, Life Technologies, Paisley, UK), and the oral fluids, nasal swabs, and bronchial lavages were diluted in phosphate saline buffer (10% *w*/*v* or *v*/*v*) both supplemented with an antibiotic (1000 U/mL penicillin, 1 mg/mL streptomycin; Gibco, Life Technologies, Paisley, UK) and an anti-mycotic (2.5 µg/mL amphotericin B; Gibco, Life Technologies, Paisley, UK). After centrifugation, RNA was extracted using a Biosprint 96 One-For-All Vet Kit (Qiagen, Hilden, Germany) on a Thermo Fisher Scientific KingFisher Apex Purification System (Waltham, MA, USA), according to the manufacturer’s instructions. Extracted RNA was analysed to detect the most common viruses involved in pig respiratory problems, particularly the swine influenza virus (SIV) [13] and porcine reproductive and respiratory syndrome virus (PRRSV) (AgPath-ID^TM^ NA and EU PRRSV Multiplex© Applied Biosystems, Waltham, MA, USA).

### 2.2. Virological Methods

The presence of porcine respirovirus 1 was determined using a real-time RT-PCR gene-HN screening method, as previously described [14]. In addition, a PRV1-positive sample with a Ct value of 32.2 was inoculated on cell culture monolayers, newborn pig trachea (NPTR), rhesus monkey kidney (LLC-MK2), and foetal monkey kidney (MARC-145) cell lines to allow for its isolation. After 2 h at 37 °C in a humidified 5% CO_2_ incubator, the inoculum was removed, and minimum essential medium (MEM) supplemented with 1% penicillin-streptomycin and 2 µg/mL of trypsin was added. The inoculated cell cultures were observed daily for 5–7 days for the appearance of a cytopathic effect (CPE). Then, they were further subcultured to improve the chance of isolating PRV1 until the fourth passage, at which moment, even in the absence of a CPE, they were tested again with PRV1-specific real-time RT-PCR.

### 2.3. Genetic Characterisation

PRV1-positive samples were subsequently characterised by Sanger sequencing using RT-PCR to amplify the entire F gene [15]. The PCR products were purified using the QIAquick PCR Purification Kit (Qiagen, Hilden, Germany). Sequencing reactions were performed with a BigDye Terminator v3.0 kit (Applied Biosystems, Lennik, Belgium) and analysed with an ABI Prism 3730 DNA Analyser (Applied Biosystems). Phylogenetic analysis was performed by comparing the obtained sequences with other PRV1 sequences available in GenBank and aligning them using ClustalW. A phylogenetic tree was constructed using the Maximum Likelihood method and the General Time Reversible model (G + I), identified using ModelFinder selection, with bootstrap analysis (1000 replicates) using MEGA10 software. The HRV1 F-gene sequence (GenBank accession: NC003461) was used as an outgroup.

Nucleotide homology percentages were determined via NCBI BLASTn analysis.

## 3. Results

**Figure 1:**
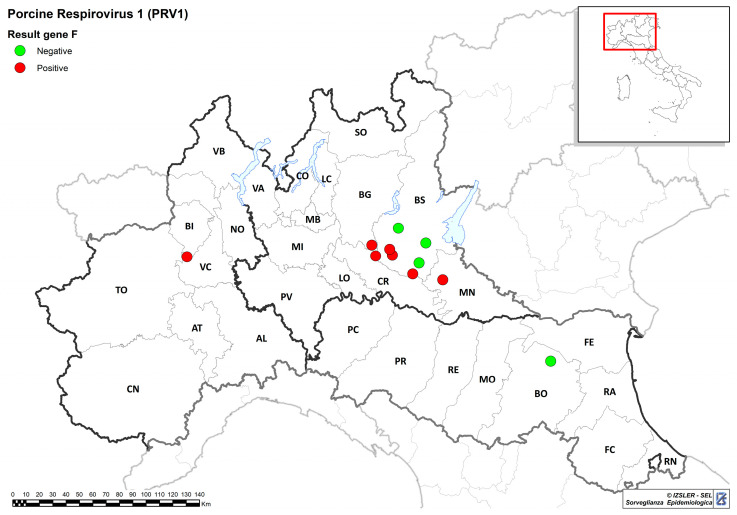
Geographical distribution of the pig farms where PRV1 was identified. Farms for which F gene sequencing was obtained or not are shown with red and green dots, respectively.

Using real-time RT-PCR to examine 902 pig samples, we identified 14 (1.55%) PRV1-positive samples: three from oral fluids (3/63, 4.76%) and 11 from the lungs, nasal swabs, and bronchial lavages (11/839, 1.31%). With reference to the origin, PRV1 was detected in 12 samples from nine farms in Lombardy, one from Emilia-Romagna, and one from Piedmont. The geographical locations of the positive farms are shown in Figure 1. In one farm, pigs of different ages (sow and pre-fattening) resulted as positive for two consecutive years (2022 and 2023). Animals of all ages resulted as positive. The clinical signs in sampled alive pigs were mainly coughing and nasal discharge, whereas in carcasses of necropsied pigs, different patterns of bronchopneumonia and pleuropneumonia were observed. Bacteriological examinations revealed a rather wide variety of bacterial species including *Streptococcus suis*, *Haemophilus parasuis*, *Mycoplasma hyorhinis*, *Salmonella choleraesuis*, *Actinobacillus pleuropneumoniae*, and *E. coli.*

**Figure 2:**
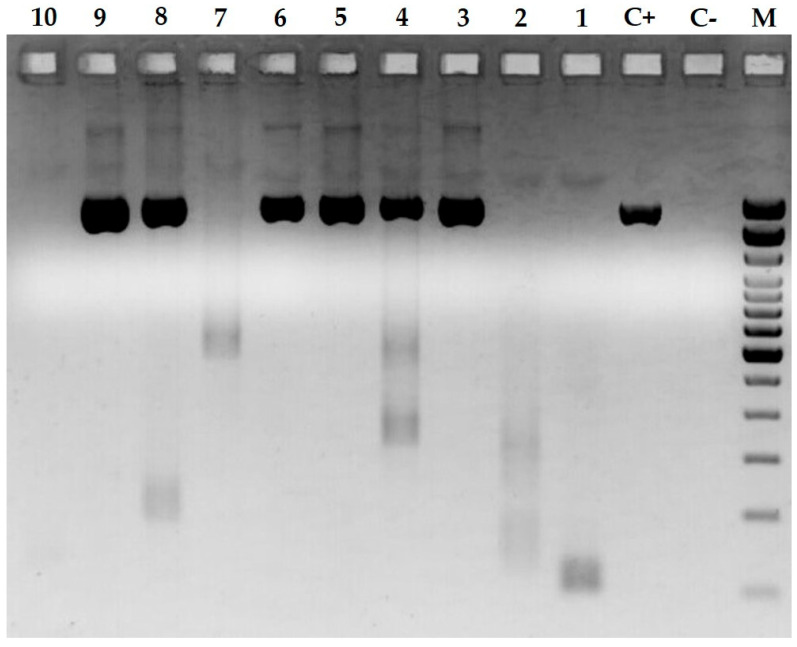
Two percent agarose gel showing the end-point RT-PCR amplification product of ten samples tested. M, marker 100 bp DNA Ladder (Invitrogen Inc., Carlsbad, CA, USA). From these results, it can be seen that samples 3, 4, 5, 6, 8, and 9 tested positive for the F-gene of interest. Samples 1, 2, 7, and 10 were negative.

The PRV1 real-time RT-PCR yielded Ct values ranging from 32 to 40 (Table 1). The 87691/2023 sample with the highest level of PRV1 viral RNA (real-time RT-PCR Ct value of 32.2) was selected for viral isolation. At 5–7 days post-infection (dpi), culture supernatants were frozen at −20 °C and passaged thrice. No CPE was observed in any of the passages, and the cryolysate from the last passage was screened via PRV1-specific real-time RT-PCR; however, it was negative. Of the 14 samples that tested positive on screening, the F sequence was obtained for 10 samples, nine of which were complete and one incomplete (Figure 2).

**Figure 3:**
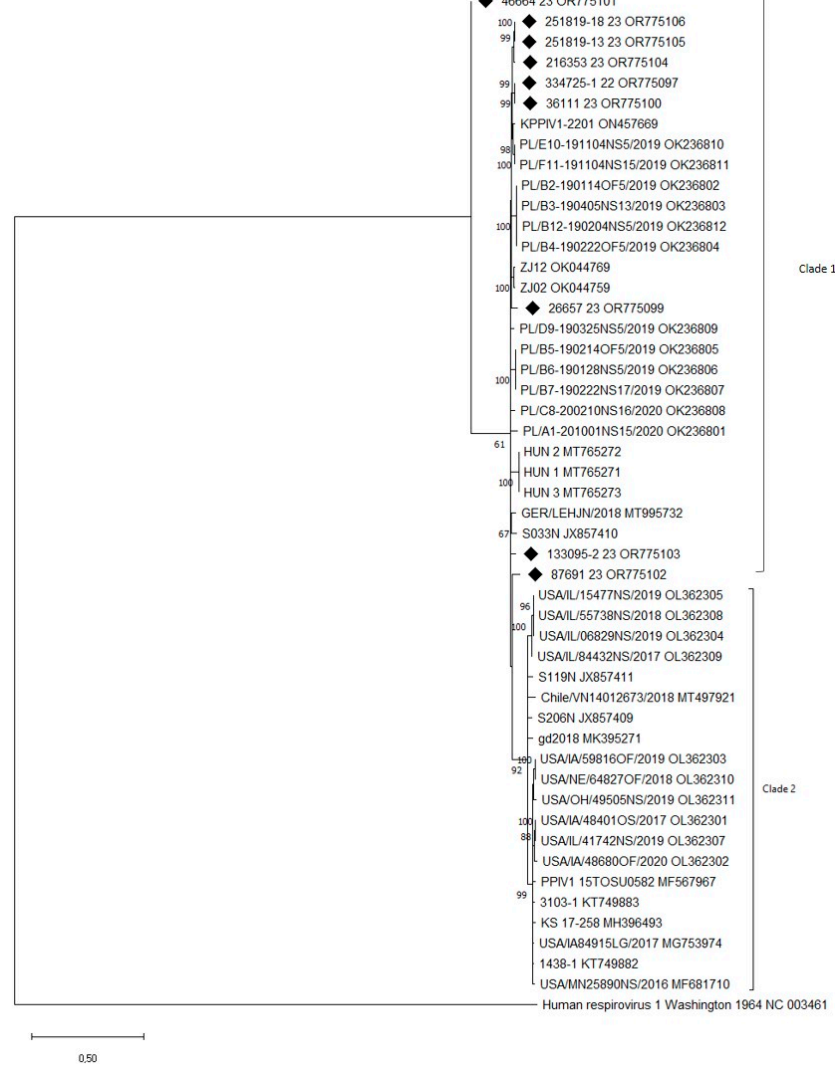
The phylogenetic tree is based on the F-gene sequencing of the PRV1s detected in the survey and the viral strains deposited in GenBank. Molecular analyses were performed with MEGA 10 software with bootstrap analysis (1000 replicates) using the Maximum Likelihood method based on the General Time Reversible + G + I model. Bootstrap values > 60% are shown. Sequences generated in this study are indicated with a black rhombus. Note that the F sequence of one strain (334725-2/2022) was excluded since it was incomplete. Published sequences are identified by strain and GenBank accession number. The F gene sequence of HRV1 (GenBank acc.: NC003461) was used as an outgroup.

The phylogenetic tree constructed on the region confirmed that the sequences clustered with PRV1 strains were previously identified in Europe and Asia (Figure 3). The 334725-2/2022 sequence was excluded because it was incomplete. All sequences obtained in this study clustered in clade 1, according to the classification of Stadejek et al. [16]. They grouped the complete F-gene sequences available in GenBank into two distinct clades with high statistical support. Clade 1, European lineage 1, contained sequences from Poland, Hungary, Germany, South Korea (KPPIV1-2201_ON457669), Hong Kong (S033N/2009_JX857410), and the Chinese strain ZJ. Clade 2 contained sequences of American lineage 2, comprising American, Chilean, and Chinese sequences.

NCBI BLASTn analysis revealed that five of the ten PRV1 sequences (334725-1/2022, 36111/2023, 216353/2023, 251819-13/2023, and 251819-18/2023) had the highest homology (96.77–97.59%) with the KPPIV1-2201 strain identified in South Korea (2022), three sequences (26657/2023, 87691/2023, and 46664/2023) with Chinese strains ZJ12 and ZJ02 (GenBank OK044769.1 and OK044759. 1), while sequence 334725-2/2022 revealed the highest identity with the Poland PL/E10 strain identified in 2019, and sequence 133095-2/2023 with the Hungarian strain HUN_4 (96.65% homology) identified in 2022.

Notably, PRV1 was always associated with either PRRSV or SIV, except in four samples (28.6%). Co-infection with SIV and PRRSV was detected in one (7.1%) and nine (64.3%) samples, respectively (Table 1). The sequences obtained in this study are available in GenBank under the accession numbers OR775097-OR775106 (Table 1). Finally, routine aerobic and anaerobic bacterial cultures revealed different isolates, including *Streptococcus suis*, *Mycoplasma hyorhinis*, *Haemophilus parasuis*, and *Actinobacillus pleuropneumoniae.*

## 4. Discussion

It is widely recognised that PRV1 is widely spread. However, pigs with PRV1 natural infection usually display mild respiratory diseases or no clinical signs. Welch et al. [17] reported that PRV1 shedding, and replication occurred under experimental conditions in the upper and lower respiratory tracts of commercial nursey-age and caesarean-derived/colostrum-deprived (CDCD) pigs. However, no significant clinical respiratory disease was observed. Other studies reported that PRV1 could cause minor sneezing, coughing, and serous nasal discharge in naturally infected pigs [7].

Our study analysed clinical samples, including lung tissues, nasal swabs, bronchial lavages, and oral fluids from conventional pigs from farms in northern Italy exhibiting respiratory signs, including sneezing, coughing, and nasal discharge, using real-time RT-PCR for PRV1 detection. Necropsy revealed mild-to-moderate macroscopic pulmonary lesions. Additionally, SIV and PRRSV infections were investigated. Data from the bacteriological examinations were also retrieved. Of the 902 samples examined, 14 (1.55%) were positive for PRV1 and were genetically characterised. The positive results for oral fluids (4.76%) confirmed the validity of this type of sampling for detecting PRV1.

Interestingly, PRV1 was detected twice on one farm in 2022 and 2023, suggesting a likely spread and persistent virus circulation within the farm, tending to make the PRV1 infection endemic. Considering the peculiarities of pig production systems in Italy, which are characterised by multiple transfers of pigs and long production cycles, both endemic and emerging pathogens can rapidly and easily spread in commercial pig farms, resulting in severe economic consequences.

Phylogenetic analysis based on the F gene [16] confirmed that the sequences obtained in this study were grouped into European lineage 1. However, sequencing other genomic regions should confirm classification at the genotype level. Unfortunately, PRV1 has not been isolated from field samples, and further studies are required. Li et al. [18] described the best in vitro culture conditions for PRV1 isolation, in which the temperature at 32 °C and trypsin addition are ideal for the growth of PRV1 in LLC-MK2 cells. In contrast, Park et al. [15] successfully isolated two PRV1 strains in LLC-MK2 cells at 37 °C. We tried isolation at 37 °C and not at 32 °C; however, we assume that virus isolation can succeed with the sample containing a higher viral RNA load. Since in all the positive samples, the Ct value was high (between 32 and 40), the lack of viral isolation was likely due to the presence of few and/or non-vital virions. Considering that the samples were all taken from pigs with respiratory signs and/or lesions, and most of them were positive for secondary bacterial infections, it could be assumed that the PRV1 infection had occurred a long time before.

Indeed, cell lines that enhance infectivity express endo-proteases such as furin, and the presence or absence of genetic factors correlates with the virus strain’s virulence. It would be interesting to study the mechanisms involved in PRV1 fusion. Whether virulent strains influence the severity of the clinical disease remains to be clarified.

In this study, the SIV and PRRSV co-infections were frequent; thus, the contemporaneous presence of PRV1 with these viruses that were implicated in respiratory problems in pigs confirmed their potential involvement in the development of the Porcine Respiratory Disease Complex (PRDC). In addition, bacterial agents that are often involved in PRDC, such as *Mycoplasma hyorhinis*, *Haemophilus parasuis*, *Streptococcus suis*, and *Actinobacillus pleuropneumoniae*, were detected in association with viral agents in this study. However, considering that the four cases in which PRV1 was the sole viral agent identified showed similar signs, it remains to be clarified whether PRV1 has a synergistic effect in infected pigs, leading to a more severe clinical form and increased mortality.

Since PRV1 sequences from Hungary, Germany, the Netherlands [10], and parts of those from Italy are closely related to the strains found in China, it is likely that a single event of transmission of PRV1 between Europe and China could have occurred, or also that these strains were subsequently established in Central Europe. Further investigation is needed to determine how widespread PRV1 is in Europe and assess its importance in PRDC. Specific molecular tests will help implement customised plans for detecting and controlling PRV1 infection.

## 5. Conclusions

Here, we assessed the presence of PRV1 in Italy, an important pig-producing area. In particular, the investigation was conducted by collecting samples from pigs with clinical signs of respiratory disease, in which the involvement of respiratory viral pathogens was suggested.

Overall, our findings permitted the detection of PRV1 in some cases of PRDC, sometimes in association with other viral agents (PRRSV and SIV). However, it remains to be clarified whether genetically different PRV1 strains showing specific virulence factors can determine and influence, possibly as a co-factor in association with other infectious agents, the onset of clinical disease or the severity of clinical signs of PRDC.

## Figures and Tables

**Table 1:** Overview of the data available for PRV1-positive samples; * the superscript number indicates samples from the same farm.

Sample ID	Sample Type	Date of Sampling	Region of Origin *	Class of Age	Clinical Signs/Lesions	Bacteriological Investigations	Real-Time RT PCR PRV-1 ^1^ (Ct Value)	RT-PCR Gene F	Co-Infection		Best BLAST		GenBank Accession Number
									SIV ^2^	PRRSV ^3^	Reference	Identity (%)	
334725-1/2022	Nasal Swab	10/2022	Lombardy ^1^	Sow	Coughing, nasal discharge	np ^5^	37.02	Pos	No	Yes	KPPIV1-2201 (South Korea)	97.59	OR775097
334725-2/2022	Nasal Swab	10/2022	Lombardy ^1^	Sow	Coughing, nasal discharge	np ^5^	36.9	Pos	No	Yes	PL/E10 (Poland)	98.75	OR775098
26657/2023	Lung	01/2023	Piedmont	4–6 weeks	Moderate catarrhal bronchopneumonia cyanosis of the skin	*Streptococcus suis*	36.4	Pos	No	No	ZJ12 (China)	95.50	OR775099
36111/2023	Lung	02/2023	Lombardy ^1^	Pre-fattening	Fibrinous pleuropericarditis	*Haemophilus parasuis Mycoplasma hyorhinis*	40.52	Pos	No	Yes	KPPIV1-2201 (South Korea)	97.41	OR775100
46664/2023	Lung	02/2023	Lombardy	4–6 weeks	Apical catarrhal bronchopneumonia, pleuritis	*Salmonella* *choleraesuis*	34.9	Pos	No	Yes	ZJ02 (China)	96.30	OR775101
87691 2023	Lung	03/2023	Lombardy	4–6 weeks	Catarrhal bronchopneumonia, fibrinous polyserositis	*Streptococcus suis* *Mycoplasma hyorhinis*	32.2	Pos	No	Yes	ZJ12 (China)	95.11	OR775102
357570-1/2022	Oral Fluid	11/2022	Lombardy	Fattening pig	na ^4^	np ^5^	39.67	Neg	No	Yes	na ^4^	na ^4^	na ^4^
104364-1 /2023	Oral Fluid	04/2023	Emilia- Romagna	Gilt	na ^4^	np ^5^	38.06	Neg	Yes	No	na ^4^	na ^4^	na ^4^
125716 /2023	Lung	04/2023	Lombardy	4–6 weeks	Moderate apical catarrhal bronchopneumonia	*Streptococcus suis*	34.67	Neg	No	Yes	na ^4^	na ^4^	na ^4^
171315 /2023	Lung	05/2023	Lombardy	4–6 weeks	Fibrinous pleuropneumonia	*Actinobacillus* *pleuropneumoniae*	36.31	Neg	No	Yes	na ^4^	na ^4^	na ^4^
133095-2 /2023	Oral Fluid	04/2023	Lombardy	Gilt	na ^4^	np ^5^	37.2	Pos	No	No	HUN_4 (Hungary)	96.65	OR775103
216353/23	Lung	07/2023	Lombardy	4–6 weeks	Bilateral bronchopneumonia in the apical, middle and diaphragmatic lobes	*Streptococcus suis* *E. coli*	35.23	Pos	No	Yes	KPPIV1-2201 (South Korea)	96.77	OR775104
251819-13/23	Nasal Swab	08/2023	Lombardy ^2^	1–4 weeks	Coughing, nasal discharge	Neg	37.33	Pos	No	No	KPPIV1-2201 (South Korea)	97.46	OR775105
251819-18/23	Nasal Swab	08/2023	Lombardy ^2^	1–4 weeks	Coughing, nasal discharge	Neg	38.07	Pos	No	No	KPPIV1-2201 (South Korea)	97.46	OR775106

^1^ PRV-1, Porcine Respirovirus 1; ^2^ SIV, Swine Influenza Virus; ^3^ PRRSV, Porcine Reproductive and Respiratory Syndrome Virus; ^4^ na, not available; ^5^ np, not performed.

## Data Availability

The data presented in this study are available on request. The sequences obtained in this study are available in GenBank under accession numbers.
